# Traumatismes fermés et pénétrants de l'abdomen: analyse rétrospective sur 175 cas et revue de la littérature

**DOI:** 10.11604/pamj.2015.20.129.5839

**Published:** 2015-02-16

**Authors:** Raherinantenaina Fanomezantsoa, Rakotomena Solonirina Davidà, Rajaonarivony Tianarivelo, Rabetsiahiny Lalao Fabienne, Rajaonanahary Toky Mamin'Ny Aina, Rakototiana Felantsoa Auberlin, Hunald Francis Allen, Rakoto Ratsimba Hery Nirina

**Affiliations:** 1 Service de Chirurgie Viscérale et Vasculaire, CHU-JRA, Antananarivo; 2 Service des Urgences, CHU-JRA, Antananarivo, Madagascar; 3 Service d'Urologiqie-Andrologie, CHU-JRA, BP 4150, Antananarivo; 4 Service de Chirurgie Viscérale Pédiatrique, CHU-JRA, BP 4150, Antananarivo

**Keywords:** Abdomen, chirurgie, contusion, plaie pénétrante, polytraumatisme, Abdomen, surgery, contusion, penetrating wound, polytrauma

## Abstract

Les traumatismes abdominaux sont relativement fréquents mais graves dans les pays en développement. Le but de cette étude était de décrire les aspects épidémiologiques, diagnostiques, thérapeutiques et évolutifs des contusions et plaies pénétrantes de l'abdomen prises en charge dans un pays à faibles ressources. Patients et méthodes: Il s'agissait d'une étude rétrospective et descriptive de 2 ans (2011-2012) ayant colligé 175 cas de traumatisés abdominaux au CHU-JRA Tananarive Madagascar. Parmi ces blessés (144 hommes et 31 femmes), il existait 122 vivants (69,7%) et 53 décès (30,3%) avant tout geste thérapeutique hospitalier. Les étiologies étaient dominées par les accidents à responsabilité civile (52,5%) et de la voie publique (38,5%). Les contusions et plaies pénétrantes représentaient respectivement 41,8% et 58,2%. Parmi les blessés vivants, 112 ont été opérés (91,8%). L’évolution hospitalière était favorable dans 94,3%. Quatre patients avaient des suites opératoires compliquées (3,6%). Sept patients étaient décédés (5,7%). Parmi les décès préhospitaliers, nous avons observé 73,6% de polytraumatisme (n = 39) et 26,4% de traumatismes abdominaux isolés (n = 14). A l'autopsie, les lésions abdominales étaient hémorragiques dans 94,3% incluant des plaies vasculaires rétropéritonéales, des ruptures hépatospléniques et des traumatismes graves du bassin. En situation précaire, les traumatismes abdominaux ont une mortalité préhospitalière assez importante. A l'hôpital, l’évolution était généralement favorable au prix d'un acte opératoire invasif.

## Introduction

Les blessures par violences ou accidents représentent la deuxième cause de mortalité dans le monde [1]. Cette mortalité est en rapport direct avec la gravité des traumatismes et le retard à la prise en charge [2]. Dans les centres experts, de nombreuses publications américaines portant sur des grandes séries de traumatismes abdominaux font état de la situation épidémiologique et de différentes attitudes diagnostiques et thérapeutiques. En France, ces traumatismes sont moins fréquents et les attitudes diagnostiques et thérapeutiques sont sensiblement différentes [3]. Le traitement optimal des blessés est établi grâce à une collaboration multidisciplinaire qui débute dès la phase préhospitalière [1]. A l'hôpital, une prise en charge non chirurgicale est actuellement privilégiée mais elle doit respecter des critères précis dont l'expérience de l’équipe médicochirurgicale et la disponibilité d'un plateau technique performant sont les éléments majeurs [4]. En situation précaire, la mortalité préhospitalière est élevée. La prise en charge des blessés est modifiée du fait de l'inadéquation des plateaux techniques et des moyens d'investigation performants qui font défaut. Le traitement non opératoire serait difficile à concrétiser et il faut adapter la stratégie diagnostique et thérapeutique aux moyens humains et techniques disponibles [2]. L'objectif de notre travail était de décrire les aspects épidémiologiques, diagnostiques, thérapeutiques et évolutifs de ces traumatismes abdominaux observés dans un pays à faibles ressources.

## Méthodes

**Critères d'inclusion et d'exclusion**: il s'agissait d'une étude rétrospective et descriptive des contusions et plaies pénétrantes de l'abdomen observées au CHU-JRA (Tananarive-Madagascar) du 01/01/2011 au 31/12/2012. Nous avons inclus les blessés vivants à l'admission et ceux décédés dans la phase préhospitalière, ayant eu une contusion ou plaie pénétrante de l'abdomen. Nous avons exclu les blessés non hospitalisés ou opérés dans d'autres centres.

**Prise en charge des blessés vivants à l'admission**: le diagnostic de plaie pénétrante était porté soit cliniquement devant une éviscération ou épiplocèle, soit radiologiquement, soit après une exploration sous anesthésie locale. Le diagnostic de contusion est évoqué devant une douleur ou écorchure abdominale avec notion de traumatisme à point d'impact abdominal, basithoracique ou pelvien. Un examen clinique complet était préalablement effectué. La mise en place d'une double voie veineuse périphérique était systématique. Les examens d'imagerie disponibles étaient les radiographies standards, l’échographie et la TDM. La prescription de ces examens était très limitée. L’échographie abdominale était surtout demandée devant un traumatisme fermé à hémodynamique stable ou stabilisé. Les radiographies standards n’étaient pas demandées devant une plaie abdominale avec éviscération ou épiplocèle. L'administration de sérum antitétanique était systématique en cas de plaie. L'intervention chirurgicale était indiquée et réalisée par des chirurgiens généralistes et/ou spécialisés. Elle était établie à partir des arguments cliniques avec ou sans examen complémentaire d'imagerie. L'indication opératoire était l'existence d'un état de choc hémorragique ou d'une instabilité hémodynamique et devant une péritonite. La laparotomie était systématique pour toute plaie pénétrante. La surveillance armée était réservée aux traumatismes fermés avec stabilité hémodynamique, sans péritonite ni pneumopéritoine. Une salle d'opération était utilisable. L'antibiothérapie préopératoire était systématique, au plus tard dès l'induction. Les plaies hépatiques étaient suturées ou faisaient appel à un hémostatique absorbable (Surgicel^®^) ou à un packing qu'on a enlevé au bout de 72h. La splénectomie était réalisée devant toute plaie de la rate. Le traitement des plaies coliques comportait une suture simple ou résection anastomose avec ou sans stomie de protection. Les plaies iléales étaient traitées par une simple suture ou résection anastomose. La cavité péritonéale était lavée et drainée en fin d'intervention. Les patients opérés étaient hospitalisés en salle de réanimation chirurgicale avant d’être transférés en chirurgie viscérale. L'antibiothérapie postopératoire était systématique. Une fiche de surveillance décrivant les paramètres vitaux était établie pour chaque patient. La réalimentation orale était autorisée dès la reprise de transit. Le drain péritonéal, une fois non productif, était enlevé au bout de trois à cinq jours. Les patients étaient systématiquement revus un mois après leur sortie de l'hôpital. Les vaccinations contre le pneumocoque étaient systématiques après splénectomies.

**Autopsie des blessés décédés dans la phase préhopsitalière**: la réalisation de l'autopsie était orientée par le contexte traumatique et la constatation de lésions externes, suivie d'une laparotomie et thoracotomie, éventuellement associée à une craniotomie.

**Paramètres étudiés**: les paramètres suivants étaient analysés: l’âge, le sexe, l’étiologie, le temps écoulé dans la phase préhospitalière, le diagnostic, le traitement, la durée d'hospitalisation, l’évolution ainsi que les causes de décès préhospitaliers.

**Méthode statistique**: les données ont été rédigées sur des cahiers d'observation, des comptes rendus opératoires et des fiches d'anesthésie-réanimation. Les comptes rendus d'autopsie précisant les causes de décès ont été consultés. Ces informations manuscrites recueillies de façon prospective ont été photographiées puis saisies dans une base de données sous forme de tableau Excel^®^

## Résultats

**Bilan général**: nous avons retenu 175 cas de traumatismes abdominaux sur 72125 dossiers enregistrés, soit 2,4 cas pour 1000 patients reçus aux urgences. En 2011 et 2012, il existait respectivement 84 et 91 victimes. A l'accueil, nous avons reçu 122 blessés vivants (69,7%) et 53 cadavres (30,3%). L’âge moyen était de 30,4 ans (1-84ans). Il y avait 144 hommes (82,3%) et 31 femmes (17,7%). Le sex-ratio était de 4,6. Les accidents à responsabilité civile (ARC) représentaient 51,4% et ceux de la voie publique (AVP) 42,3%. Les plaies et contusions de l'abdomen représentaient respectivement 52,6% et 47,4%. Le traumatisme était isolé dans 77,1% et multiple dans 22,9%. Chez les décès préhospitaliers, nous avons noté 39 cas de polytraumatisme et 14 cas de traumatisme abdominal isolé (Tableau 1). Les lésions après traumatismes fermés étaient secondaires à un accident de voiture dans 85,6% (n = 71), chute accidentelle dans 3,6% et accident de moto dans 2,4%. Sept patients vivants à l'admission se sont déclarés victimes de coups et blessures (8,4%). Pour les 92 cas de plaies pénétrantes, il s'agissait des lésions par arme blanche dans 58 cas, arme feu dans 25 cas et encornement de zébu dans 9 cas. Parmi les blessés par arme blanche, 46 cas étaient victimes d'agressions (79,3%), 10 ont eu un accident de travail (17,2%) et 2 ont été blessés par autolyse (3,5%). Les blessés vivants à l'admission avaient un délai de prise en charge inférieur à 6h dans 83,6%, entre 6 et 24h dans 5,7% et plus de 24h dans 10,7%. Le délai moyen était de 13h (1-168h). Pour les décès préhospitaliers, le temps écoulé entre l'accident et l'hôpital était de 2,7h en moyenne (1-4h).

**Tableau 1:** Répartition des blessés vivants à l'admission et des décès préhospitaliers

Paramètres	Poucentage (%)	Effectif(n)	Blessés vivants à l'admission (n) (%)	Décès pré hospitaliers (n) (%)
Effectif		175	122 (69,7)	53 (30,3)
Sexe				
Homme	82,3	144	105 (86,0)	39 (73,6)
Femme	17,7	31	17 (14,0)	14 (26,4)
ARC	51,4	90	64 (52,5)	26 (49,0)
AVP	42,3	74	47 (38,5)	27 (51,0)
Accident de travail	5,1	9	9 (7,4)	0 (00,0)
Autolyse	1,2	2	2 (1,6)	0 (00,0)
Polytraumatisme	22,9	40	1 (0,8)	39 (73,6)
Traumatisme isolé	77,1	135	121 (99,2)	14 (26,4)
Traumatismes fermés	47,4	83	51 (41,8)	32 (60,4)
Plaies pénétrantes	52,6	92	71 (58,2)	21 (39,6)
Arme à feu	27,2	25	11 (15,5)	14 (66,7)
Arme blanche	63	58	51 (71,8)	7 (33,3)
Encornement de zébu	9,8	9	9 (12,7)	0 (00,0)

ARC: accident à responsabilité civile; AVP: accident de la voie publique

### Blessés vivants à l'admission

**Clinique**: l'examen clinique trouvait 5 cas de choc hémorragique et un cas de choc septique. Après contusions, nous avons retrouvé une instabilité hémodynamique avec péritonite dans 19 cas (37,3%), stabilité hémodynamique et péritonite dans 24 cas (47%) et stabilité hémodynamique sans péritonite (asymptomatique) dans 8 cas (15,7%). Après plaies pénétrantes, les blessés étaient hémodynamiquement instables dans 26,1% et stables dans 51,1%. La répartition des orifices d'entrée cutanés figure sur le Tableau 2. L’épiplocèle était retrouvée chez 12 patients (20%). Les blessés par arme blanche et encornement de zébu étaient hémodynamiquement stables dans 42 cas (70%) et instables dans 18 cas (30%). Parmi eux, il existait 24 cas de péritonite (40%), un cas de choc hémorragique et 29 cas de blessés asymptomatiques (48,3%). Parmi les 11 cas de plaies par arme à feu, il existait 5 cas de stabilité et 6 cas d'instabilité hémodynamique soit 3 patients asymptomatiques et 8 cas de péritonite.

**Tableau 2:** Répartition des orifices cutanés d'entrée chez les blessés vivants

Orifices cutanés d'entrée	Plaies par arme blanche	Plaies par arme à feu	Plaies par encornement de zébu
Hypochondre droit	7	0	1
Hypochondre gauche	4	2	2
Epigastre	14	3	0
Epigastre + Fosse iliaque droite	0	1	0
Epigastre + hypochondre droit	1	0	0
Flanc droit	3	0	1
Flanc gauche	8	2	0
Péri ombilical	4	1	1
Fosse iliaque droite	1	0	1
Fosse iliaque gauche	4	0	1
Hypogastre	1	0	0
Fosse lombaire droite	2	1	0
Fosse lombaire gauche	0	0	0
Basi thoracique droit	0	0	0
Basi thoracique gauche	0	1	0
Péri-anal	2	0	2
**Total**	**51 (71,8%)**	**11 (15,5%)**	**9 (12,7%)**

**Paraclinique (imagerie)**: après contusions, la radiographie standard était réalisée chez 5 patients avec image de pneumopéritoine dans 4 cas. Aucun examen tomodensitométrique n’était réalisé. Quinze patients ont effectué des examens échographiques (29,4%). Trente et un patient ne pouvaient pas bénéficier d'examens paracliniques (60,8%). Les blessés par arme blanche et encornement de zébu avaient 7 fois des examens radiologiques conventionnels avec image de pneumopéritoine (11,7%). Cinquante-trois patients ne pouvaient pas bénéficier d'examen complémentaire (88,3%). Cinq patients avaient des examens échographiques. Aucun patient n'a bénéficié de TDM. Après plaies par arme à feu, 8 patients n'avaient aucun examen radiologique. Les 3 autres avaient des examens radiographiques (n = 3), échographique (n = 1) et tomodensitométrique (n = 1).

**Figure 1:**
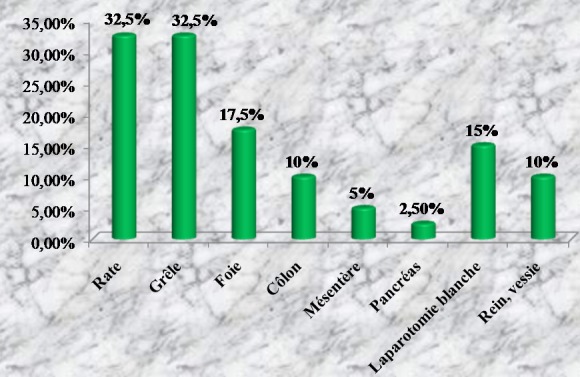
Lésions viscérales après traumatismes fermés

**Figure 2:**
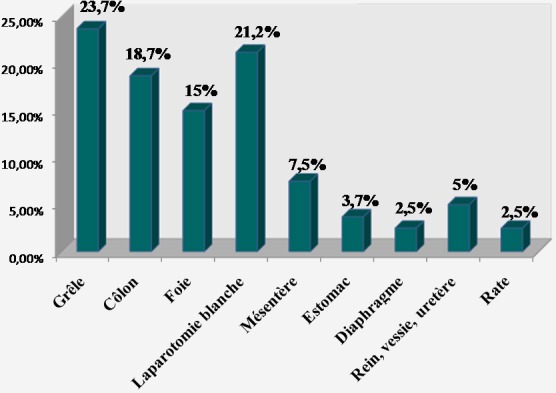
Lésions viscérales par arme blanche

**Figure 3:**
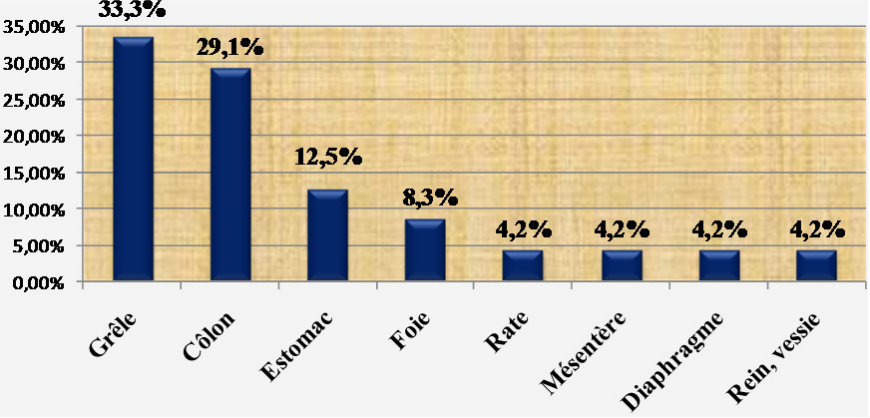
Lésions viscérales par arme à feu

**Figure 4:**
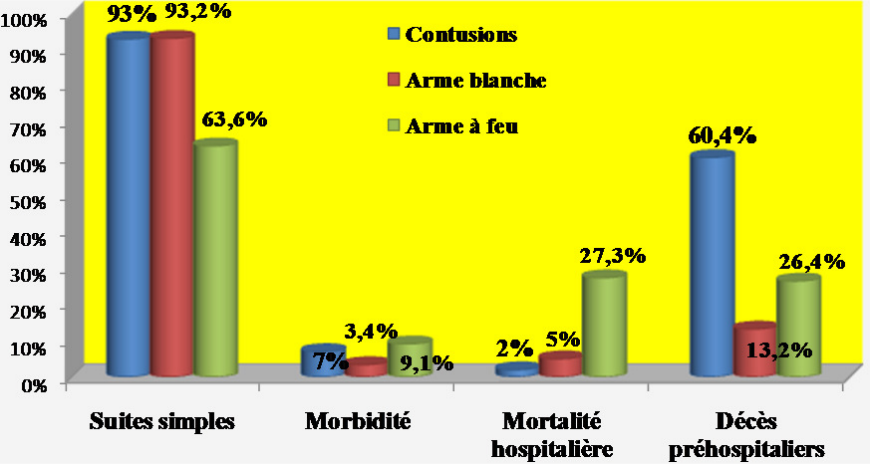
Évolutions de tous les cas étudiés

**Traitement**: à part les remplissages vasculaires par des macromolécules, les patients à hémodynamique instable avaient en moyenne deux poches de sang total et/ou de culot globulaire (1-3). Chaque patient ayant eu une plaie abdominale avait reçu une dose de sérum antitétanique. En périopératoire, les blessés avaient une double antibiothérapie à dose optimale (ceftriaxone + métronidazole). Au total, 112 patients étaient opérés (91,8%) et 8 traités de façon non opératoire (6,6%). Deux patients ne pouvaient pas être opérés. Aucun patient n'a subi de laparotomie écourtée. Les plaies pénétrantes étaient opérées sauf un cas décédé en salle de déchoquage. Parmi les patients victimes de traumatismes fermés, 42 étaient directement opérés après leur passage aux urgences (82,4%). Un patient, décédé avant induction au bloc opératoire, était autopsié. Huit patients ont bénéficié d'une surveillance armée. Ils étaient tous cliniquement asymptomatiques. Toutes les explorations chirurgicales étaient menées par laparotomie médiane. En moyenne, la durée opératoire était de 2h (1-5h). L'ensemble des lésions viscérales observées après traumatismes fermés est détaillé dans la Figure 1. Il y avait 32,5% de lésions spléniques et 17,5% de plaies hépatiques. La laparotomie était blanche ou non thérapeutique dans 15%. Les blessés par arme blanche et encornement de zébu avaient des lésions viscérales dont la répartition est représentée sur la Figure 2. Nous avons trouvé 23,7% de lésions iléales et 18,2% de plaies coliques. La laparotomie était blanche dans 21,2%. La répartition des lésions viscérales par arme à feu est détaillée dans la Figure 3. Les lésions étaient dominées par les plaies iléales (33,3%) et coliques (29,1%). Il n'y avait pas de laparotomie blanche. Les gestes chirurgicaux effectués sont détaillés dans le Tableau 3. Les 8 cas non opérés étaient victimes de traumatismes abdominaux fermés, stables sur le plan hémodynamique et n'ayant présenté aucun signe de péritonite ni de pneumopéritoine. Ils ont tous bénéficié d'un hémogramme. A l’échographie, trois patients n'avaient aucune lésion viscérale. Les 5 autres avaient un hémopéritoine de faible abondance en rapport avec un hématome sous capsulaire de la rate et du foie. La durée moyenne de séjour hospitalier était de 8j (1-21) pour les traumatismes fermés et 7j (1-15) pour les plaies pénétrantes. Les données sur l’évolution sont présentées sur la Figure 4. Avec un recul moyen de 10 mois, 90,2% des patients n'avaient aucune complication. Aucun patient n’était perdu de vue. L’évolution globale était favorable dans 94,3%. Le taux de mortalité hospitalière était de 5,7% (n = 7). Il s'agissait des patients décédés en préopératoire par choc hémorragique (n = 2) et en postopératoire précoce (n = 5) par choc septique et défaillance multiviscérale. Aucun décès n’était survenu en peropératoire. Les suites opératoires étaient simples dans 93% des cas chez les blessés par contusions abdominales. Trois patients avaient des suites compliquées (7%) dont deux abcès de paroi et une occlusion intestinale. Les deux cas d'abcès étaient secondaires à des lésions iléo-coliques compliquées de péritonite en préopératoire. L'occlusion intestinale était observée chez un patient splénectomisé 3 mois après l'intervention initiale. Le patient décédé à l'admission avait une rupture de la rate découverte à l'autopsie. Les blessés par arme blanche et encornement de zébu ont eu des suites opératoires simples dans 93,2% (n = 55). Deux patients ont eu des suites opératoires compliquées (3,4%): une coagulopathie intra-vasculaire disséminée et une occlusion intestinale. La coagulopathie était découverte en peropératoire et l'occlusion était secondaire à des adhérences retrouvées 3 mois après une laparotomie blanche. Trois patients étaient décédés (5%) dont un en préopératoire par choc hémorragique et deux en postopératoire précoce par sepsis et défaillance viscérale. Les deux derniers cas avaient une péritonite stercorale préopératoire d'origine iléo-colique. Le cas autopsié était un blessé vivant à l'admission mais décédé de complications hémorragiques. A l'autopsie, nous avons découvert une plaie vasculaire mésentérique. Parmi les blessés par arme à feu, 7 patients avaient des suites simples, un cas développait une suppuration pariétale et les 3 autres étaient décédés par choc hémorragique (n = 1) et septique (n = 2). En moyenne, le rétablissement de continuité digestive était réalisé 3 mois après l'intervention initiale. Aucune complication n'a été observée.

**Tableau 3:** Répartition et fréquence des gestes chirurgicaux effectués

Principaux gestes chirurgicaux	Traumatismes fermés (n)	Plaies par arme blanche et encornement de zébu (n)	Plaies par arme à feu (n)
**Grêle**			
**Suture**	5	14	3
**Résection anastomose**	9	6	4
**Iléostomie**	0	0	0
**Côlon**			
**Résection anastomose**	1	0	0
**Colostomie/iléostomie**	2	6	1
**Colectomie**	0	1	1
**Foie**			
**Suture**	1	10	0
**Packing périhépatique**	2	1	0
**Surgicel** ^**®**^	3	1	0
**Electrocoagulation**	0	0	1
**Rate: splénectomie**	13	2	1
**Estomac: suture**	0	3	3
**Diaphragme: suture**	0	0	1
**Mésentère: suture**	0	6	0

**Décès préhospitaliers**: l'autopsie des blessés à traumatismes fermés de l'abdomen révélait un hémopéritoine de 2,5L en moyenne. Le bilan des lésions extra-abdominales associées chez les polytraumatisés révélait 43,1% de lésions cardiothoraciques, 29,3% de lésions cérébrales et 27,6% de lésions orthopédiques. Parmi eux, il y avait trois cas de plaie aortique et trois cas de traumatismes graves du bassin. Les lésions intra-abdominales étaient hémorragiques dans 93,7% incluant des ruptures spléniques (28,1%), hépatiques (25%), des plaies vasculaires rétropéritonéales (25%) et des ruptures hépatospléniques associées (15,6%). Chez les blessés par plaies pénétrantes, l'hémopéritoine était de 3L en moyenne. Les blessés par arme blanche avaient en moyenne deux lésions viscérales. Nous avons observé des lésions vasculaires rétropéritonéales dans 26,7% (aorte abdominale, VCI, artère iliaque commune) et des plaies hépatospléniques dans 40%. Les plaies par arme à feu avaient entrainé 3 lésions viscérales par victime. Il s'agissait des plaies hépatiques (34,8%), gastro-intestinales (26%), spléniques (8,6%) et de la VCI (8,7%).

## Discussion

D'après cette étude les traumatismes abdominaux n’étaient pas fréquents: ils ne représentaient que 0,24% des patients reçus aux urgences chirurgicales. Ce résultat était toutefois assez modeste compte tenu de la monocentricité de l’étude. Néanmoins, nous avons constaté une augmentation de leur fréquence, probablement en rapport avec le développement anarchique de la circulation automobile et des agressions à main armée. L'adulte jeune était la population cible avec un âge moyen aux alentours de 30 ans. Ce pic de fréquence correspond au stade de la vie où les actes médico-légaux par délinquance ou violence sont fréquents dans notre pays. Deux études rétrospectives sur les plaies de l'abdomen ont retrouvé un âge moyen de 27,3 à 36 ans [3, 5]. Ces résultats ont été respectivement observés en Afrique et en France. Quant aux traumatismes fermés, un âge moyen de 27 ans était rapporté en milieu défavorisé [6]. Dans le même contexte, une étude malgache publiée en 2008 rapportait un âge moyen de 29 ans chez 316 blessés par accident de circulation [7]. Les cas rapportés intéressaient aussi bien les hommes que les femmes avec une prédominance masculine. Il s'agit d'une situation classique constamment observée dans de nombreuses publications. Les étiologies varient d'un pays à l'autre en fonction des contextes économiques, culturels et sociopolitiques. Le plus souvent, elles sont représentées par les ARC et AVP. Cette étude ne fait que les confirmer. Les autres étiologies sont rares: accident de travail, tentative de suicide. L'encornement de zébu, responsable de plaies abdominales dans notre série, correspondait à un accident de travail car les attaques évoquées étaient observées lors de la riziculture. Aux États-Unis, l’étiologie est plus fréquemment violente, par arme à feu ou par arme blanche (60%) [4]. En Europe et hors des zones de guerres, les traumatismes abdominaux surviennent dans plus de 60% des cas au cours d'accidents sur la voie publique [8]. En France, la majorité des traumatismes abdominaux sévères survient lors des accidents de la voie publique. Un rapport récent de l'OMS décrit les traumatismes de la voie publique comme une épidémie gravissime, car ils sont responsables dans le monde de 1,2 million de décès par an: en effet, alors que la mortalité par AVP tend à diminuer en Europe et aux Etats-Unis. Elle ne fait qu'augmenter dans les pays moins riches du fait du développement anarchique de la circulation automobile (90% des décès par AVP dans le monde surviennent chez des blessés habitant des pays à revenu faible ou moyen) [9]. En l'occurrence, nous avons rencontré 42% de décès préhospitaliers dus aux AVP. La plupart des blessés étaient polytraumatisés.

Dans cette étude, le délai de prise en charge était, dans la majorité des cas, inclus dans les 6 premières heures (83,6%). Ce qui n'est pas loin des résultats rapportés dans d'autres pays à faibles ressources [5–7]. Cependant, le délai moyen était assez important (13h) puisqu'il n’était pas rare de se retrouver devant des cas vus tardivement. Les raisons sont multiples: distance entre le lieu de l'accident et l'hôpital, manque de transport médicalisé, méfait de la médecine traditionnelle. Pour les décès préhospitaliers, le temps écoulé entre l'accident et l'hôpital était de 1 à 4h. Ce délai plus ou moins tardif reflète la gravité de ces blessés qui font précipiter les entourages vers l'hôpital au moyen des transports, le plus souvent, de fortune. Car les secours sur les lieux de l'accident étaient quasi inexistants, inefficaces et tardifs. En termes d'urgence, la mortalité occasionnée par les traumatismes abdominaux dépend de la qualité et de la précocité de la prise en charge préhospitalière. Un délai plus long peut entrainer la mort chez les polytraumatisés ou victimes de lésions hémorragiques non contrôlées dans cette phase. Un délai plus court occasionne le même sort à cause de l'effet hémorragique et hypoxique des lésions gravissimes. La mort peut survenir juste après le traumatisme, en cours de transport ou exceptionnellement avant l'acte opératoire. En situation précaire, le plateau technique est souvent défavorisé. Il existe une inadéquation entre les besoins et les moyens disponibles en personnels, examens paracliniques et possibilités thérapeutiques [2]. La prise en charge pose de difficiles problèmes diagnostiques car l'examen clinique est souvent non contributif [4]; or l'indication opératoire dans cette situation est souvent basée sur les résultats de cet examen, le bilan d'imagerie étant souvent incomplet. Devant un traumatisme fermé, l'appréciation de l’état hémodynamique et la recherche d'une défense abdominale sont l’élément clé de la décision thérapeutique. Dans notre série, la prédominance de péritonite est expliquée par le retard diagnostic des plaies iléo-coliques et de l'abondance de l'hémopéritoine secondaire à des lésions hépatospléniques. Le taux élevé de laparotomie blanche (15%) correspondait à une mauvaise sélection des patients peu symptomatiques dont l'indication opératoire était essentiellement fondée sur l'existence d'un hémopéritoine décelable à l’échographie. Les patients symptomatiques blessés par arme blanche méritaient une exploration chirurgicale.

Cependant, il y avait 48,3% de patients asymptomatiques qui n'auraient pas dus être opérés en urgence. Puisque en rapportant ce résultat clinique aux lésions viscérales observées en peropératoire, nous avons constaté un taux assez important de laparotomie blanche (21,2%). Par contre, après plaies par arme à feu, aucune laparotomie blanche n'a été rapportée même chez les patients asymptomatiques. Ainsi, quelque soit le résultat de l'examen clinique nous plaidons au maintien du dogme de la laparotomie systématique devant toute plaie pénétrante par arme à feu. Compte tenu de la précarité de notre plateau technique, beaucoup de patients ne pouvaient pas bénéficier d'examens radiologiques adaptés. Pourtant, l’échographie abdominale, la radiographie du thorax et du bassin sont de pratique courante dans la prise en charge de ces blessés. Elles peuvent être pratiquées dans les 30 minutes suivant l'arrivée du blessé à l'hôpital et orientent vers un traitement qui est approprié dans 98% des cas [10]. Le scanner spiralé, exceptionnellement faisable dans notre série, possède une importance croissante pour le diagnostic [1]. Les examens radiologiques standards étaient rarement demandés pour des raisons économiques, financières et contextuelles. Dans la littérature, les radiographies d'ASP, du thorax et les clichés centrés sur les coupoles sont très peu contributifs, sauf en cas d'hémopéritoine ou de pneumopéritoine massifs, et ne doivent plus faire partie des examens morphologiques des patients victimes d'une plaie de l'abdomen [4]. En alternative, l’échographie d'urgence, échographie pleurale, péricardique et péritonéale (E3P), est très largement pratiquée. Elle est d'une grande sensibilité pour la mise en évidence des épanchements liquidiens, voire gazeux, et peut être mise en œuvre dès la phase préhospitalière [11]. Dans cette étude, la laparotomie était réalisée de façon systématique pour les plaies pénétrantes afin de ne pas laisser évoluer à bas bruit une plaie initialement asymptomatique du tube digestif conduisant à une péritonite de pronostic sévère. Après contusions, le taux de laparotomie blanche observé dans notre série (15%) correspondait aux données de la littérature (5 à 16,6%) [7, 12]. Pour les plaies par arme blanche, la prédominance des lésions iléales (22,4 à 27,8%) était une situation classique; le taux de laparotomie non thérapeutique égal à 21,2% étant inférieur aux résultats observés dans d'autres pays (24 à 40%) [5, 13]. Les plaies par arme à feu entrainent fréquemment des lésions iléales et coliques, en raison de la nature agressive de cet agent vulnérant et du fait de la topographie anatomique et de l’étendu de l'espace que ces viscères occupent dans la cavité abdominale. Dans la phase préhospitalière, l’évolution et le pronostic des patients gravement blessés dépendent de la rapidité et de l'efficacité des traitements instaurés. L'absence de premiers soins sur les lieux de l'accident, le manque de transport médicalisé et l'inadéquation des soins au niveau des structures d'accueil ne permettent pas de contrôler précocement l'hémorragie ou de prévenir l'aggravation des lésions extra-abdominales associées. Dans notre étude, 4% des blessés vivants à l'admission étaient en état de choc hémorragique. La majorité des blessés graves étaient décédés avant leur admission aux urgences (30,3%).

En Afrique, une étude rapportait 6,8% de décès préopératoires dans un état de choc irréversible [5]. En France, les décès préopératoires sont devenus rares grâce au développement de la transfusion massive et de la laparotomie écourtée. Dans les centres experts, plus de 90% des traumatisés de l'abdomen sont stables hémodynamiquement à leur admission grâce au progrès de la réanimation préhospitalière. Environ, seulement 5% des blessés sont admis en état grave, avec nécessité de réanimation. Face à ce type de blessé le plus souvent instable, qui saigne, les objectifs sont de prévenir la survenue de la triade létale de Moore (acidose, hypothermie et coagulopathie) et de limiter les risques infectieux [14]. Le protocole permettant de maitriser ces complications est actuellement bien codifié [1]. Le remplissage vasculaire fait appel aux solutés cristalloïdes et au sérum salé hypertonique. La lutte contre l'hypothermie passe par le monitorage continu de la température centrale, le réchauffement externe et interne passif. L'antibioprophylaxie visant en particulier les entérobactéries et les germes anaérobies est administrée de façon systématique. Le traitement de la coagulopathie comprend l'administration précoce de produits dérivés du sang. En urgence, la laparotomie reste la méthode de choix pour sauver les blessés instables hémodynamiquement ou présentant une péritonite; les autres options (embolisation, laparoscopie) étant réservées aux centres experts. L'abord laparoscopique, qui est loin d’être pratiqué dans notre centre, est régulièrement discuté depuis plus de 20 ans pour la prise en charge des plaies pénétrantes de l'abdomen du patient stable, sans éviscération ni péritonite [1]. Elle limite le nombre de laparotomies blanches, mais ne permet pas l'exploration exhaustive des viscères intra-abdominaux. Le traitement non opératoire (TNO) a été exceptionnellement adopté et n’était possible que chez quelques patients bien sélectionnés. Il s'agit d'une attitude thérapeutique non invasive et conservatrice [4] dont l'objectif étant d’éviter les laparotomies inutiles et de diminuer la morbimortalité opératoire. Ce TNO a été adopté par beaucoup d'auteurs pour prendre en charge les lésions rétropéritonéales, hépatiques et/ou spléniques afin d'améliorer leur pronostic [4]. Mais cette stratégie ne peut être envisagée que dans un milieu adapté avec un monitoring attentif, des examens cliniques répétés, la possibilité de transfusions massives, un plateau technique diagnostique et interventionnel ainsi qu'un bloc opératoire prêt à tout instant à une laparotomie urgente [4]. L'existence de lacune en termes de faisabilité pour l'imagerie et les difficultés d'assurer un TNO étaient à l'origine de notre attitude thérapeutique invasive. Toutefois, elle nous a offert une prise en charge correcte en milieu défavorisé avec les techniques habituelles de réparation viscérale, mise en place de packing et splénectomie totale. Son avantage était de découvrir précocement les lésions digestives. Si le traitement chirurgical des lésions gastroduodénales et de l'intestin grêle est bien codifié et privilégie les réparations immédiates, le traitement des lésions coliques est plus controversé. Il a évolué depuis une dizaine d'années vers une attitude d'emblée réparatrice au détriment de dérivations, qui sont réservées aux patients présentant des facteurs de haut risque de fistule. Les motifs de ces réparations immédiates sont confortés par le taux de complications liées à la fermeture secondaire de la colostomie: occlusion ou abcès profonds et fistules. En Afrique, la durée d'hospitalisation se trouve améliorée au fil du temps. En effet, elle était de 8 à 29 jours (5-216) en 1996 [5] et 10 jours en 2008 [7]. Actuellement, nous avons observé un séjour moyen de 7-8 jours (1-21). A l'origine de ce résultat, nous avons retenu trois explications: les patients gravement blessés étaient décédés en préhospitalier, les lésions traitées à l'hôpital étaient majoritairement bénignes, le séjour hospitalier était réduit après laparotomie blanche. En France, une étude rapportait un séjour moyen de 7 jours (1-21) pour les blessés par arme blanche et 20 jours (1-83) pour ceux par arme à feu [3]. Dans cette étude, le long séjour était lié aux modalités thérapeutiques telles qu'une surveillance armée suivie de laparotomies et à la gravité des lésions par arme feu dont les suites opératoires étaient émaillées de complications morbides ayant nécessité de réparations chirurgicales itératives. Malgré la précarité de notre plateau technique, nous avons eu des suites opératoires majoritairement simples. A l'origine de ce bon résultat, nous avons noté plusieurs facteurs: hémostase et réparation chirurgicale correctes, antibioprophylaxie systématique, durée opératoire relativement courte, réalisation de stomie et alimentation parentérale systématique chez les patients à haut risque de fistule, prévention des complications thromboemboliques et infectieuses chez les splénectomisés. Selon les études, une chirurgie précoce et adaptée limite significativement la mortalité, mais aussi la morbidité des traumatismes pénétrants abdominaux. Avec un recul moyen de 10 mois, seuls quelques patients ont eu des suites compliquées, avec une morbidité faible. Cependant, cette morbidité peut aller jusqu’à 25% en milieu très défavorisé. Elle est souvent observée dans la prise en charge des plaies pénétrantes [5] comme cela avait été le cas dans notre étude. En situation précaire, l’évolution des traumatismes abdominaux n'est pas sans risque de décès hospitaliers. En effet, 4,5% de nos patients opérés décédaient dans les 4 premiers jours. Les causes de décès étaient d'ordre infectieux par contamination intra-abdominale secondaire à des perforations coliques. A Lomé, la majorité des décès opératoires (10,8%) étaient secondaires à des complications hémorragiques apparues dans les 48 premières heures [5]. Dans les centres qui disposent d'un plateau technique performant, les décès opératoires par choc hémorragique sont très rares. Il est rapporté que le taux de mortalité et de morbidité postopératoire est surtout lié aux lésions coliques qui sont les plus contaminantes [4]. Le plus souvent, cette mortalité est due au sepsis intra-abdominal et à la défaillance multiviscérale. La mortalité hospitalière globale des traumatismes pénétrants de l'abdomen est évaluée à 8%, respectivement 2% et 16% par arme blanche et arme à feu [3]. En France, la mortalité hospitalière des plaies abdominales par arme blanche reste élevée, entre 10 et 15%. A la phase initiale, elle est due aux complications hémorragiques majeures. Les décès à la phase secondaire sont en relation avec les complications infectieuses, telles qu'abcès, péritonite ou encore gangrène gazeuse [1]. Notre étude rapportait 8,4% de mortalité périopératoire après plaies pénétrantes. La cause de mortalité était dominée par la survenue d'un sepsis intra-abdominal, secondaire à des péritonites stercorales préopératoires. Ce qui fait que l'antibioprophylaxie est toujours indispensable car l'infection est la principale complication des traumatismes abdominaux pénétrants. Elle survient dans 10 à 15% des cas. Parmi eux, la mortalité attribuable est d'environ 30% [15]. Pour les plaies viscérales hémorragiques, les lésions hépatiques constituent la première cause de mortalité par contusion de l'abdomen. Ces lésions sont à l'origine de décès précoces indus, le plus souvent par hémorragie. Dans notre série, 38,2% des décès préhospitaliers avaient de ruptures hépatiques, secondaires à des traumatismes fermés. Dans les pays avancés, cette mortalité intéressait 15% des blessés dont 20 à 25% sont en rapport direct avec les lésions hépatiques elles-mêmes [16].

Chez les blessés graves, le polytraumatisme (AVP) était l'une des causes de décès préhospitaliers (73,6% dans notre étude). Le plus souvent, il survient lors des accidents de la voie publique [4]. Les associations lésionnelles aggravant le pronostic des blessés étaient d'ordre neurologique, orthopédique et cardiothoracique. Ces lésions entrainent des répercussions sur les fonctions vitales, mais aussi les unes par rapport aux autres. Dans les premières minutes, ce sont l'hémorragie et l'hypoxie qui tuent les blessés [2]. Les plaies pénétrantes ont la réputation d’être toujours mortelle à cause de leur gravité immédiate extrême en cas d'atteinte des gros vaisseaux, du foie ou de la rate. La mortalité globale par ces traumatismes en préhospitalier est de 15% (6% par arme blanche et 32% par arme à feu) [17]. Cette mortalité augmente en cas de plaie thoraco-abdominale, chez le sujet âgé, en cas de retard à la prise en charge thérapeutique, de traumatisme crânien associé. La mortalité est généralement supérieure à 50%, due à l'hémorragie massive et aux lésions associées [18]. Il a été rapporté que les plaies de gros vaisseaux sont rapidement mortelles en absence d'hémostase rapide. Elles sont souvent secondaires à des traumatismes pénétrants (88 à 95%) et les facteurs de mauvais pronostic sont le caractère central des lésions, leur multiplicité ou leur association à d'autres lésions et l'existence d'un état de choc [18]. Ces caractéristiques lésionnelles expliquent la gravité supérieure des traumatismes par arme à feu. Cependant, une plaie vasculaire intra-abdominale par arme blanche peut être aussi de mauvais pronostic comme en témoignent les cas autopsiés dans notre série (aorte abdominale, VCI, artère iliaque commune). Ces traumatismes vasculaires ont une mortalité supérieure à 50% dont 15% avant tout geste chirurgical [18]. Les traumatismes abdominaux sévères concernent parfois plusieurs régions anatomiques. Les plaies thoracoabdominales sont les plus fréquentes et sont associées à une mortalité élevée supérieure à 30%. Leur prise en charge nécessite dans 20% des cas une thoracotomie de sauvetage soulignant la gravité de ces patients dès la prise en charge [19]. Le traitement chirurgical de ces patients instables peut être rapidement compromis par la survenue au bloc opératoire, mais parfois dès la phase préhospitalière, d'un cercle vicieux associant acidose métabolique, hypothermie et coagulopathie. Son incidence est surtout corrélée à la gravité des patients, mais aussi à la durée de la chirurgie. Dans notre étude, les lésions intra-abdominales responsables du tableau hémorragique pour les décès préhospitaliers, étaient des plaies hépatospléniques, des lésions vasculaires rétropéritonéales et des traumatismes graves du bassin. Selon une étude [20], les traumatismes vasculaires et les hématomes rétropéritonéaux sont responsables d'une mortalité de 42 à 67%. Les différentes causes des décès étaient: défaillance multiviscérale (34,8%), traumatisme crânien (21,8%), embolie pulmonaire (6,5%), traumatisme thoracique (4,3%), désamorçage cardiaque (28,2%), syndrome du compartiment abdominal (4,4%). La population décrite dans cette étude était jeune et majoritairement masculine et comporte plus de 90% de traumatismes fermés. La principale cause de perte de chance de ces patients gravement blessés était un retard à la réalisation de la laparotomie.

## Conclusion

Ces traumatismes de l'abdomen constituent un réel problème de santé publique dans le monde, en Afrique et surtout dans les pays à faibles ressources comme le notre. Cette étude a permis d'avoir un aperçu sur les particularités épidémiologiques des traumatismes abdominaux en milieu défavorisé. Il s'agit de la première série des cas rapportés à Madagascar prenant en charge de façon plus ou moins comparative les contusions et plaies de l'abdomen dans une même rubrique. Dans cette étude, ces traumatismes n’étaient pas fréquents mais graves car ils sont responsables d'une mortalité préhospitalière assez importante. Les facteurs de mauvais pronostic sont les AVP, le polytraumatisme et les lésions par arme à feu. A l'hôpital, l’évolution était généralement favorable avec une morbidité faible au prix d'un acte opératoire invasif. En perspective, les plaies pénétrantes par arme à feu seront opérées en urgence et de façon systématique. Les plaies par arme blanche feront l'objet d'une étude prospective visant à diminuer le taux de laparotomie non thérapeutique.
